# Combination of *Vitex pseudo-negundo* methanolic-extract with cisplatin can induce antioxidant activity and apoptosis in HeLa and Caski cells

**DOI:** 10.3389/fphar.2024.1476152

**Published:** 2024-12-04

**Authors:** Parisa Shiri Aghbash, Javid Sadri Nahand, Omid Rahbar Farzam, Seyed Mohammad Reza Hosseini, Mobina Bayat, Taher Entezari Maleki, Hossein Bannazadeh Baghi

**Affiliations:** ^1^ Infectious and Tropical Diseases Research Center, Tabriz University of Medical Sciences, Tabriz, Iran; ^2^ Department of Virology, Faculty of Medicine, Tabriz University of Medical Sciences, Tabriz, Iran; ^3^ Immunology Research Center, Tabriz University of Medical Sciences, Tabriz, Iran; ^4^ School of Medicine, Zanjan University of Medical Sciences, Zanjan, Iran; ^5^ Faculty of Pharmacy, Tabriz University of Medical Sciences, Tabriz, Iran; ^6^ Cardiovascular Research Center, Tabriz University of Medical Sciences, Tabriz, Iran

**Keywords:** Vitex pseudo-negundo, cisplatin, combination therapy, human papillomavirus, cervical cancer, botanical drug

## Abstract

**Background:**

Cisplatin-based chemotherapy as a common therapeutic regimen for cervical cancer patients, is becoming more and more ineffective due to high resistance. This urges the need for introducing novel metabolics such as botanical drugs with the capacity to increase the cisplatin effectiveness. In that regard, here we investigated the anticancer effects of the Cisplatin-*Vitex pseudo-Negundo* combination in cervical cancer cell lines.

**Method and Material:**

*V. pseudo-Negundo* fruits were dried and extracted methanolic fraction. The MTT assay was performed to evaluate cytotoxicity of both drugs in CaSki and HeLa cells. Then, apoptosis, ROS production, and cell cycling were assessed by flow cytometry assay in cells treated with *V. pseudo-Negundo* and Cisplatin and their combination. Also, the rate of cell migration and colony formation were measured, using wound healing and colony formation assay, respectively. Also, the expression level of related genes (*CD133*, *BAX*, *BCL2*, *Casp-3/8/9*, *MMP-3)* was evaluated using the RT-PCR method.

**Results:**

The obtained results established that the *V. pseudo-Negundo* plant has medicinal properties to induce apoptotic and antioxidant signals. The combination treatment of methanol extraction and Cisplatin had a cytotoxic effect on cervical cancer cell lines (HeLa and CaSki) compared to monotherapy. Also, combination therapy resulted in an increased apoptosis rate and diminished ROS production in both CaSki and HeLa cell lines. Furthermore, *V. pseudo-Negundo* and Cisplatin combination therapy leads to cell cycle arrest in the G2-M and G0-G1 phase in HeLa and CaSki cell lines, respectively. Moreover, combination therapy decreased the colony formation and cell motility in both cell lines and upregulated caspases gene expression.

**Conclusion:**

The combination of *V. pseudo-Negundo* with Cisplatin therapy results in a significant anti-cancer and antioxidant effect compared to cisplatin, representing a promising candidate for future clinical investigations.

## Graphical Abstract

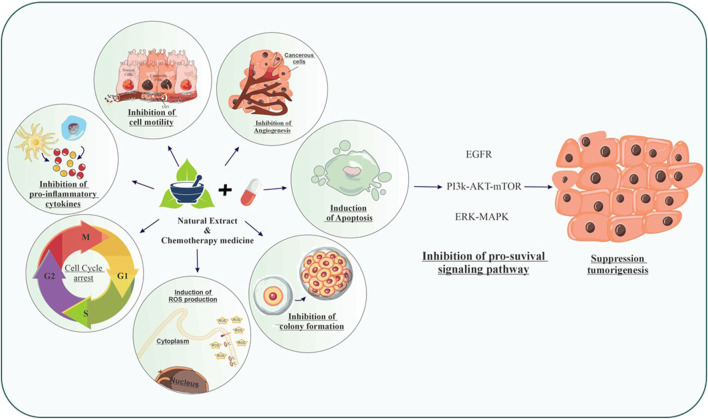

## 1 Introduction

Accounting for more than a quarter of a million annual fatalities, cervical cancer (CC) is a vital health concern worldwide (Small et al., 2017). The financial and medical burden of CC requires global efforts to achieve advancements in treatment methods. The International Federation of Gynecology and Obstetrics staging system provides the foundation for cervical cancer treatment (Tsikouras et al., 2016). Patients can get standard therapies such as surgery, chemotherapy, and radiation depending on the stage of their malignancy; however, women with metastatic cervical cancer are not yet eligible for any mainstream therapies (Li et al., 2016). Chemotherapy and preventative immunization are now the main treatment interventions for cervical cancer (Shiri Aghbash et al., 2023). Similar to other treatments, chemotherapy is faced with several limitations, including the requirement for high dosages, toxicity, and activation of pro-survival pathways after prolonged exposure, all of which result in diminished effectiveness and chemo-resistance (Florea and Büsselberg, 2011; Rastogi et al., 2015; Liu et al., 2019a). Moreover, one of the side effects of chemotherapy medicines such as Cisplatin is nephrotoxicity the related mechanism of which is not known during the use of Cisplatin. Also, currently, there is no effective methods or strategies to protect the kidneys during chemotherapy (Ahmad et al., 2019). In this regard, research indicates that the side effects caused by the use of Cisplatin, such as oxidative damage, are reduced or improved following the use of antioxidants (Almutairi et al., 2017; Darwish et al., 2018; Hazman et al., 2018). In other words, human cancer antitumor therapy frequently involves the administration of antioxidant and apoptosis-inducing medicines that prevent growth, invasion, and metastasis (Talib et al., 2022). Advances in the field of cancer treatment have become a high-profile and multi-billion-dollar industry; hence, targeted therapy has led to the reduction of limitations associated with chemotherapy (Dasari et al., 2022). Human cancer antitumor therapy frequently involves the administration of antioxidant and apoptosis-inducing medicines that prevent growth, invasion, and metastasis. Also, for decades herbal medicines have been utilized to treat various disorders and malignancies (Graham et al., 2000). Some species of *Vitex*, such as *Vitex agnus-castus*, *Vitex trifolia*, *Vitex rotundifolia,* and *Vitex negundo* can be used as herbal medicines (Jing et al., 2019). In this way, in the study conducted by Puglia et al., in 2023, it was shown that *Vitex-agnus-castus* can lead to the regulation of prolactin in individuals with mild prolactinemia (Puglia et al., 2023). Therefore, developing novel and potent anticancer substances derived from natural sources are useful for cancer treatment. This encourages further investigations to develop promising cancer treatments. In this regard, with the ultimate objective of identifying and creating new anti-CC drugs, medicinal plants have been thoroughly considered. It is also suggested that botanical drugs and natural medicines be considered in order to investigate the effectiveness of chemotherapy and reduce drug resistance (Nimlamool et al., 2022). *Vitex Pseudo-Negundo* (Hausskn.) is a plant from the Lamiaceae family and grows naturally near the seasonal rivers of Iran (Salehpour et al., 2018). This botanical drug is a traditional medicine that is used for the treatment of various diseases and disorders that affect women, including amenorrhea, dysmenorrhea, corpus luteum failure, hyperprolactinemia, infertility, acne, and menopause (Tandon and Gupta, 2005). Preclinical investigations have confirmed pharmacological roles for several of these substances. The existence of estrogenic, dopaminergic, opioids, antineoplastic, immunomodulatory, and antioxidant activities has therefore been established by several researchers (Adamov et al., 2022). Moreover, studies on phytochemistry have revealed that this genus’s members include substances such as flavonoids, terpenoids, ecdysteroids, and iridoid glycosides (Kirtikar and Basu, 1918). The *V. pseudo-Negundo* fruit extraction includes 20 flavonoids, 11 phenolic acids, 10 iridoids, 12 terpenes, and 3 diterpene alkaloids. Therefore, it can be used as an anti-cancer and antioxidant medicine in malignancies. Moreover, while many novel medicines are currently being developed in cancer treatment, combination therapies are highly anticipated. Most regions of the world have historically used natural botanical drugs for cancer treatment (Liu et al., 2019b). Natural botanical drugs have showed the capacity to elevate the efficiency of conventional chemo- and radio-therapy and promote cell death in cancer cells, prevent metastasis, as well as trigger the anticancer immunity (Liu et al., 2019b). In this regard, in 2022, Dasari et al., reported that the combined treatment of cisplatin and natural products as new strategic approaches to cancer treatment, are effective (Dasari et al., 2022). In addition, due to the existence of some bioactive metabolites in medicinal plants, they can be considered as promising sources for the treatment of various human malignancies (Dasari et al., 2022). During clinical studies, it has been established that the metabolites in medicinal plants can increase the therapeutic activity of Cisplatin as well as reduce the toxicity caused by chemotherapy. Also, metabolites found in medicinal plants modulate multiple gene transcription factors and induce cell death via apoptosis or necrosis, as a result lead to protection against organ toxicity caused by Cisplatin (Dasari et al., 2022).

Considering the need for overcoming the cisplatin-based chemo-resistance and the effectiveness of botanical drug’s metabolites for increasing the efficacy of this therapeutic regimens, here we investigated the combined effect of *Vitex* botanical medicine and cisplatin chemotherapy drug on synergistic toxicity, improving the effectiveness of chemotherapy drugs and inhibiting the drug resistance of CC cells.

## 2 Methods

### 2.1 Methanolic extraction of *Vitex pseudo-Negund*o fruit

This plant with the local name Five Fingers belongs to the Lamiaceae family. The *Vitex pseudo-Negundo* is a shrub with a height of about 3 m and is used as a medicinal species and grows naturally around the seasonal rivers and streams of Iran. After collection of the *V. pseudo-Negundo* in winter, the *V. pseudo-Negundo* fruits were dried and ground into a powder before being subjected to a soaking extraction method. In this process, an alcoholic solvent was utilized, with 100 mL employed for each extraction to yield the methanolic extract. To purify the final extract, 1 gr of the plant material underwent freeze-drying. The final *V. pseudo-Negunda* extract (3 mg of 1 gr) was dissolved in 30 μL of dimethyl sulfoxide (DMSO) (Sigma-Aldrich, United States) as a stock. Using a sensitive scale, 3 mg of the desired extract was weighed and placed in a microtube. Then about 30 μL of DMSO and 1,000 μL of distilled water were added to the extract to reduce the cytotoxicity of DMSO. The final volume of our stock reached 3 mg/mL. To reduce the toxicity effect of DMSO on the cells in the per wells, the concentration of this substance in each well was less than 1% (Aldaghi et al., 2016; Garbi et al., 2015).The obtained solution was stored on covered glass plates at −20°C, at dark, for cell culture experiments (Aldaghi et al., 2016; Gong et al., 2021). Also, according to the Heinrich et al., we speculated that our botanical drug is in the type C group (Heinrich et al., 2022).

### 2.2 Cell culture and treatment

Human cervical cancer cell lines, CaSki and HeLa, were sourced from the Iranian Biological Resource Centre (IBRC). They were cultured in a monolayer form using Roswell Park Memorial Institute medium (RPMI 1640; Gibco, United States), supplemented with 10% fetal bovine serum (FBS; Gibco, United States), along with 100 units/mL of penicillin and streptomycin in T25 flasks. The cells were maintained at 37°C in a humidified atmosphere with 5% CO2 until they reached logarithmic growth. When the cells achieved about 70% confluency, they were detached using a 0.25% Trypsin-EDTA solution (Gibco, United States) for subsequent experiments.

### 2.3 MTT assay

The 3-(4,5-dimethylthiazol-2-yl)-2,5-diphenyltetrazolium bromide (MTT) assay was performed to evaluate the viability and proliferation of CaSki and HeLa cells, determining the IC_50_ values for Cisplatin in both monotherapy and conjunction with varying concentrations of *V. pseudo-Negundo* extract (1, 10, 20, 30, 40, 50, 60, 70, 80, 90, 100, 110, 120, 130, 140, 150, 160, 170, 180, 190, 200 μg/mL). Among other treatments. Normal human foreskin fibroblast (HFF2) cells were included for comparison. Approximately 1.5 × 10^4^ CaSki and 1 × 10^4^ HeLa and HFF_2_ cells were seeded in a 96-well plate. The cell lines were divided into four treatment groups: control, Cisplatin only, *V. pseudo-Negundo* extract only, and a combination of both. After a 24-h incubation, various concentrations of the agents replaced the medium. Subsequent incubation for another 24 h was followed by adding 150 µL of MTT solution (2 mg/mL) and incubating for another 4 h at 37°C with 5% CO2. The solution was then removed, and to dissolve the resultant formazan crystals, 150 μL of DMSO was added. Absorbance at 570 nm (reference at 620 nm) was measured using a microplate reader (Tecan, Switzerland). Each assay was conducted in triplicate. Also, we examined 50% cell viability at different concentrations of both agents in both cell lines, using a [0.5:10] ratio of cisplatin to *V. pseudo-Negundo*, respectively.

### 2.4 Wound healing assay

To evaluate cell migration, an assay was performed using CaSki (3 × 10^5^ cells) and HeLa (2 × 10^5^ cells) lines after treatment with Cisplatin and *V. pseudo-Negundo* extract. Cells were plated as a singular layer in 24-well plates and incubated for 24 h at 37°C with 5% CO_2_. A linear wound was created with a 100 μL pipette, and the respective IC_50_ concentrations of the treatments were administered. Migration was tracked at three-time intervals—0, 24, and 48 hours—utilizing an inverted microscope (Optika, XDS-3, Italy).

### 2.5 Real-time PCR (RT-PCR)

For this experiment, 3 × 10^5^ CaSki cells and 2 × 10^5^ HeLa cells were cultured in 6-well plates, and divided into four treatment groups as specified earlier. Following a 24-h incubation at 37°C in a 5% CO_2_ atmosphere, cells were treated for an additional 24 h with their respective IC50 doses of Cisplatin and *V. pseudo-Negundo*. RNA extraction was executed using TRIzol reagent (RiboEX, South Korea), and RNA concentrations were quantified with a Nano-Drop spectrophotometer (Thermo Scientific, United States) at various wavelengths (230, 260, and 280 nm). Complementary DNA (cDNA) synthesis followed using the 2X RT-PCR Pre-Mix (BioFACT™, South Korea) to assess relative gene expression levels. Genes of interest included CD133, BAX, BCL2, Casp-3, Casp-8, Casp-9, and MMP-3, evaluated using the 2X Real-Time PCR Master Mix (BioFACT™, South Korea) on a StepOnePlus Real-Time PCR system (Applied Biosystems, Foster City, United States). GAPDH served as a normalization control, and analysis was performed with the 2^−ΔΔCT^ method (Shiri Aghbash et al., 2023). All primer sequences were confirmed with NCBI’s Primer-BLAST before the experiment; their sequences are presented in Table 1.

**TABLE 1:** The primer pairs sequences used for qRT-PCR.

Gene name	Forward	Reverse
CD133	5՛-GAC​CGA​CTG​AGA​CCC​AAC​ATC-3՛	5՛-GGC​TAG​TTT​TCA​CGC​TGG​TCA-3՛
BAX	5՛-TTT​GCT​TCA​GGG​TTT​CAT​CCA-3՛	5՛-TCT​GCA​GCT​CCA​TGT​TAC​TGT​C-3՛
BCL2	5՛-CTG​TGG​ATG​ACT​GAG​TAC​CTG-3՛	5՛-GAG​ACA​GCC​AGG​AGA​AAT​CA-3՛
Casp-3	5՛-CAA​ACC​TCA​GGG​AAA​CAT​TCA​G-3՛	5՛-CAC​ACA​AAC​AAA​ACT​GCT​CC-3՛
Casp-8	5՛-CGG​ACT​CTC​CAA​GAG​AAC​AGG-3՛	5՛-TCA​AAG​GTC​GTG​GTC​AAA​GCC-3՛
Casp-9	5՛-CTG​TCT​ACG​GCA​CAG​ATG​GAT-3՛	5՛-GGG​ACT​CGT​CTT​CAG​GGG​AA-3՛
MMP3	5՛-CAC​TCA​CAG​ACC​TGA​CTC​GGT​T-3՛	5՛-AAG​CAG​GAT​CAC​AGT​TGG​CTG​G-3՛

### 2.6 Apoptosis assay

In the apoptosis evaluation, 3 × 10^5^ CaSki cells and 2 × 10^5^ HeLa cells were cultured in separate 6-well plates (Shiri Aghbash et al., 2023). After 24 h, the groups were treated with their respective IC_50_ concentrations of Cisplatin and *V. pseudo-Negundo*. The Annexin V/PI staining kit (Exbio-Czech) was employed to prepare samples for flow cytometry. Cells were washed with 1X PBS and labeled with FITC-conjugated Annexin V and propidium iodide (PI) per the manufacturer’s protocol. Apoptotic cells were then quantified via flow cytometry.

### 2.7 Cell cycle assay

Following the seeding of 3 × 10^5^ CaSki cells and 2 × 10^5^ HeLa cells in 6-well plates, the cells were treated with their respective IC_50_ concentrations. After washing with PBS, cells were fixed in 70% ethanol and stored at −20°C overnight. They were then re-suspended in PBS with RNase A (200 μg/mL) and incubated at 37°C for 30 min before being stained with DAPI (50 μg/mL) for analysis. Flow cytometry (Miltenyi Biotec MACSQuant 10) was used to assess cell cycle distributions, and data were analyzed using FlowJo FACS software.

### 2.8 Assessment of cellular ROS generation

Intracellular ROS levels were evaluated using the dichloro-dihydro-fluorescein diacetate (DCFH-DA; Meilunbio, China) assay. CaSki and HeLa cells (3 × 10^5^ and 2 × 10^5^ respectively) were grown in 6-well plates. Following treatment with their respective IC_50_ concentrations, cells were washed twice with PBS in a dark setting and stained with 20 μM DCFH-DA for 30 min at 37°C.

### 2.9 Colony formation assay

For the colony formation assessment, 10^4^ CaSki cells and 5 × 10^3^ HeLa cells were placed in 6-well plates. After an incubation period of 24 h at 37°C with 5% CO_2_, cells were treated with their designated drug doses. The medium was refreshed every 2–3 days, with colony counts performed after 10–12 days. Colonies were stained with crystal violet for 30 min, and the number of colonies was quantified using ImageJ software.

### 2.10 Statistical analysis

Statistical analyses were carried out using SPSS version 26 (SPSS Inc., Chicago, IL, United States), Prism 8.0 (GraphPad, San Diego, CA, United States), and CompuSyn software. The distribution of quantitative variables was assessed through the Kolmogorov-Smirnov test. Data were presented as mean ± standard deviation (SD), with a significance level established at p < 0.05. The Wilcoxon signed-rank test or paired t-test tested paired measurements, while one-way ANOVA compared group differences.

## 3 Results

### 3.1 Combination of cisplatin and *Vitex pseudo*-*negundo* suppresses cervical cancer cells viability

**FIGURE 1:**
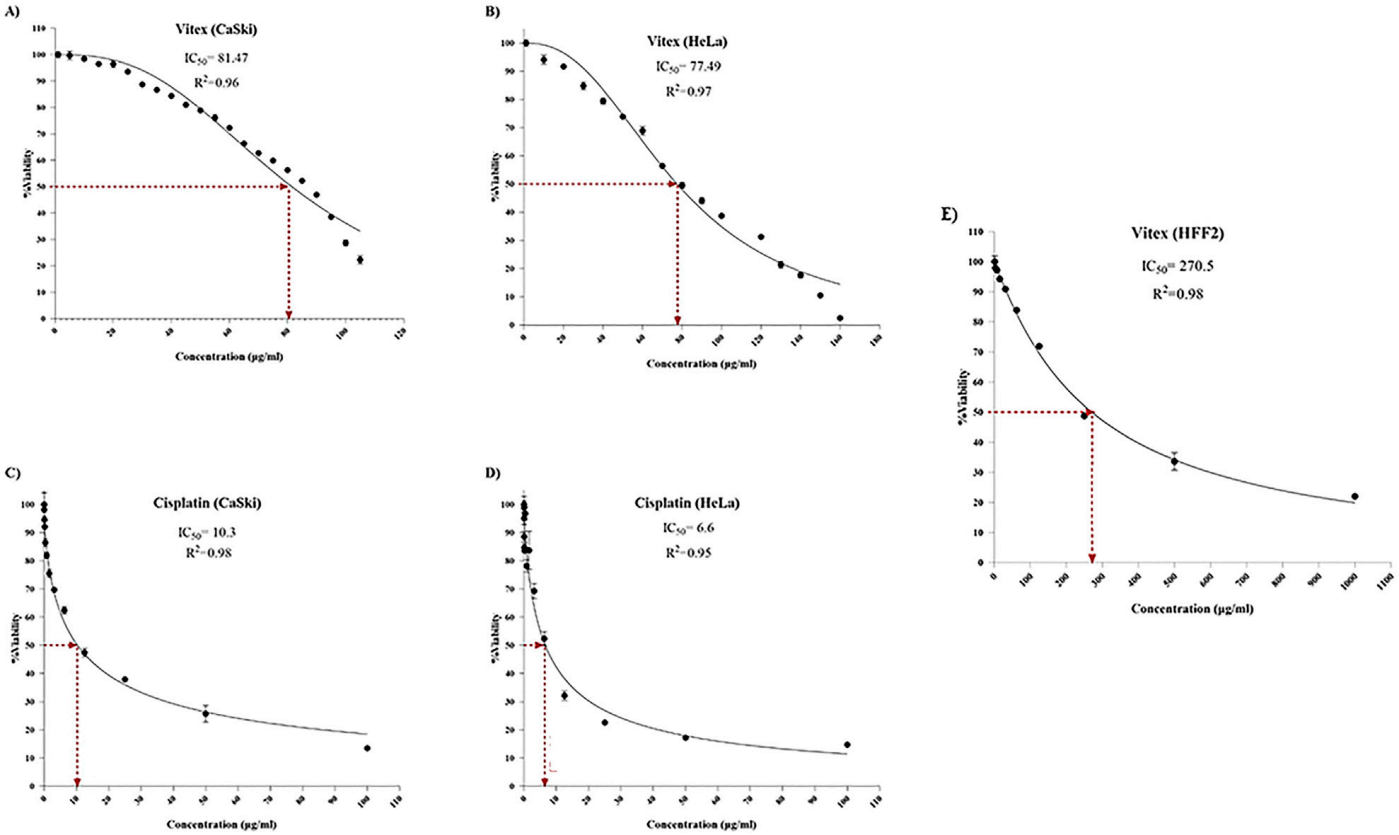
The impact of *Vitex* extraction and Cisplatin monotherapy on the viability of **(A)** *Vitex* (CaSki); **(B)** *Vitex* (HeLa); **(C)** Cisplatin (CaSki); **(D)** Cisplatin (HeLa); **(E)** *Vitex* (HFF_2_).

The treatment with Cisplatin alone resulted in approximately 50% inhibition of cell viability and proliferation in CaSki and HeLa cells at concentrations of 10.3 μg/mL and 6.6 μg/mL, respectively, compared to controls. In contrast, the extraction of *V. pseudo-Negundo* displayed a notable growth-inhibiting effect on cervical cancer cells, particularly at 81.47 μg/mL for CaSki and 77.49 μg/mL for HeLa. The application of various *V. pseudo-Negundo* extract concentrations demonstrated a significant reduction in cell viability for both cancer cell lines. Specifically, the concentrations at which cell viability in CaSki cells dropped were between 125 μg/mL and 150 μg/mL, while for HeLa cells, it ranged from 150 μg/mL to 200 μg/mL. For comparison, HFF2 cells showed an IC_50_ value of 270.5 μg/mL for *V. pseudo-Negundo* (Figure 1).

**FIGURE 2:**
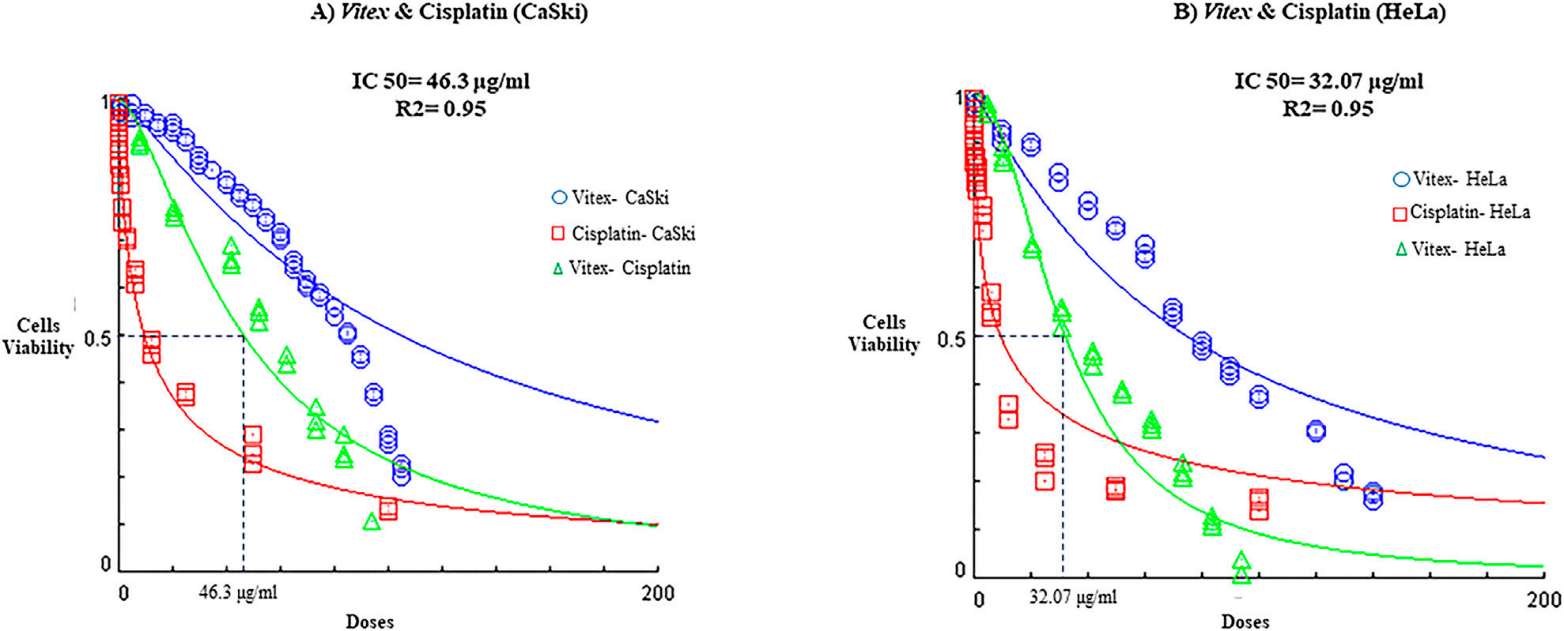
The impact of *Vitex* extraction and cisplatin combination on the viability of **(A)** CaSki cell line; **(B)** HeLa cell line.

Importantly, *V. pseudo-Negundo* extraction was found to lower the IC_50_ threshold for Cisplatin, effectively reducing the required concentration of Cisplatin to achieve less than 50% viability. The synergy between *V. pseudo-Negundo* and Cisplatin was evident, with a 10:0.5 ratio leading to IC_50_ values of 32.07 μg/mL and 42.3 μg/mL for HeLa and CaSki cells, respectively (Figure 2).

### 3.2 *Vitex pseudo*-*negundo* can sensitize cervical cancer cells to cisplatin-induced apoptosis

**FIGURE 3:**
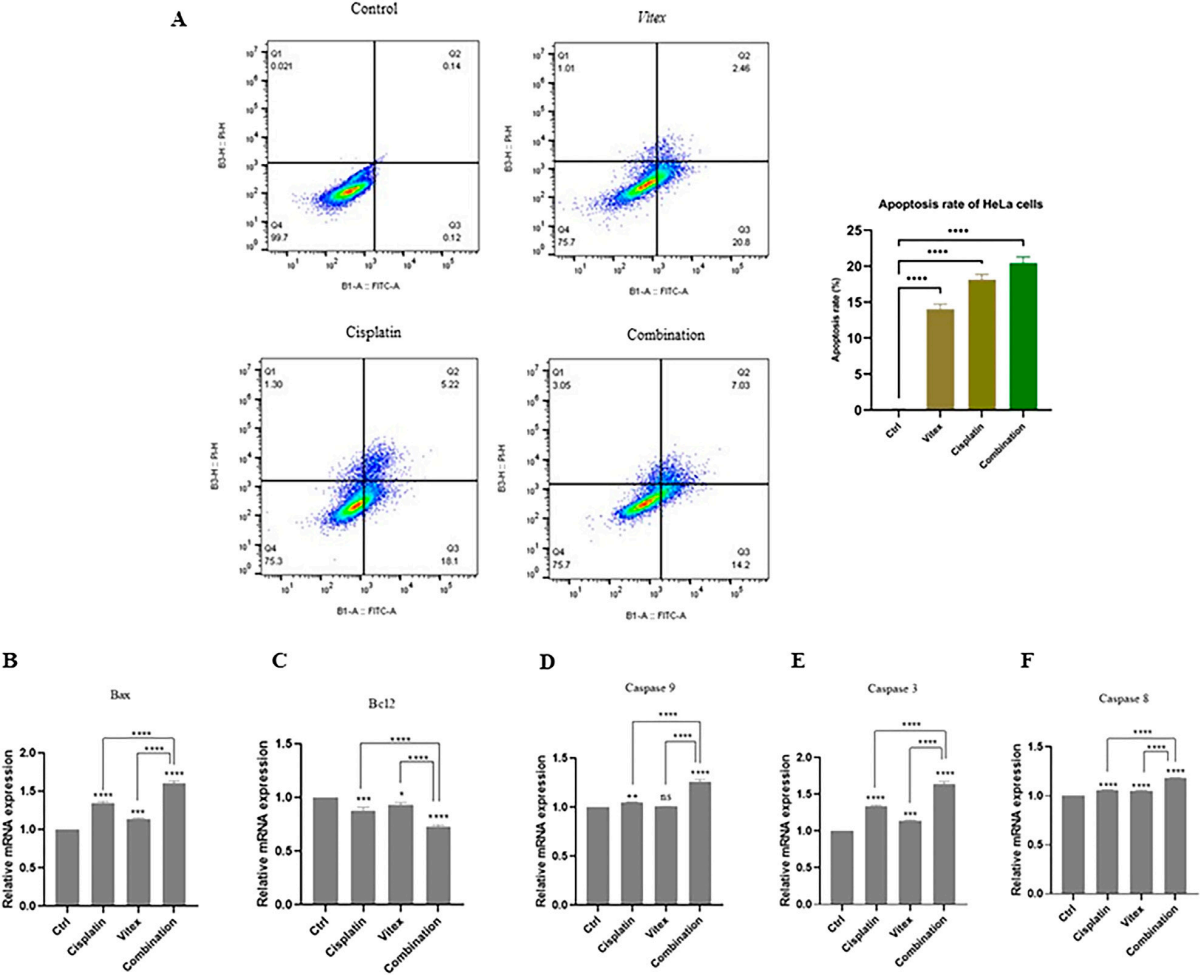
The effect of the *Vitex* extract combined with Cisplatin on apoptosis in HeLa cells. **(A)** The combination notably enhances HeLa cell apoptosis; **(B, C)** Bax levels increasing and Bcl_2_ levels decreasing compared to controls. **(D, E, F)** Caspase gene expression significantly rose following combination therapy versus monotherapy with either *Vitex* or Cisplatin (*p < 0.05, **p < 0.01, ***p < 0.001, ****p < 0.0001).

**FIGURE 4:**
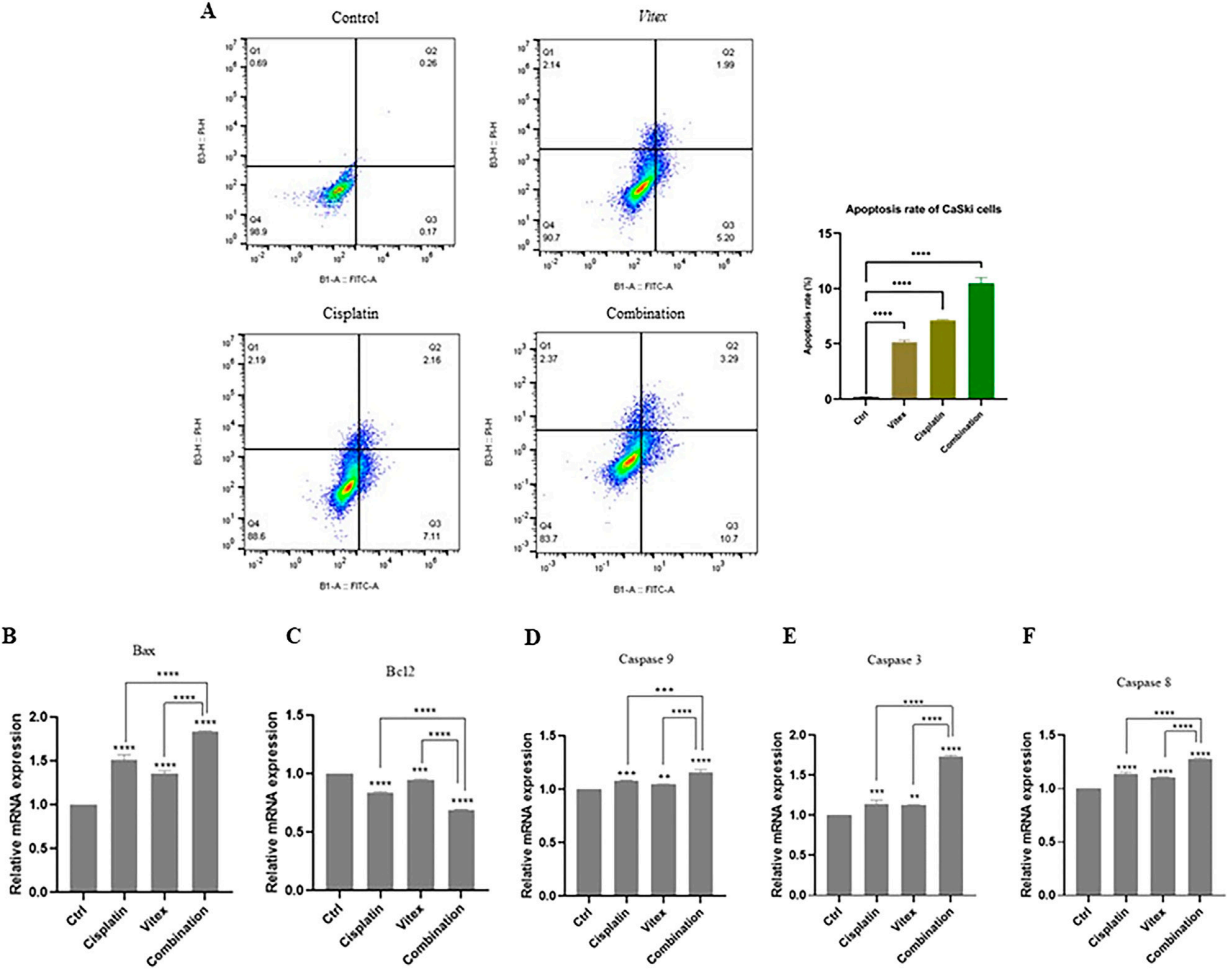
The impact of the *Vitex* extract and Cisplatin combination on apoptosis in CaSki cells. **(A)** the combination promotes increased apoptosis in CaSki cells, **(B, C)** expression level of Bax and Bcl_2_ changed, **(D, E, F)** significant rise in Caspase gene expression compared to monotherapy (*p < 0.05, **p < 0.01, ***p < 0.001, ****p < 0.0001).

Data suggest that the combination therapy led to an increased rate of apoptosis in cervical cancer cells when compared to control and monotherapy conditions. In HeLa cells, the rates of apoptosis following individual treatments of *V. pseudo-Negundo* and Cisplatin were recorded at 14.2% and 18.1%, respectively, while the combination treatment achieved a rate of 20.8%. There were no significant differences between the monotherapy and combination groups; however, each was significantly different from the controls (p< 0.0001) (Figures 3C–F). In CaSki cells, the monotherapy groups showed marked differences (*V. pseudo-Negundo:* 5.20%, Cisplatin: 7.11%) compared to the combination group (10.7%), with all treatments yielding significant differences from the control (p < 0.0001). Additionally, a remarkable increase in the expression of apoptosis-related genes such as Casp-3, -8, -9, and Bax was observed after combination therapy, contrasting with a notable decrease in the expression of the anti-apoptotic gene Bcl_2_ (Figures 4C–F).

### 3.3 *Vitex pseudo*-*negundo* extraction in combination with cisplatin arrested CaSki and HeLa cells on G0-G1 and G2-M stage

**FIGURE 5:**
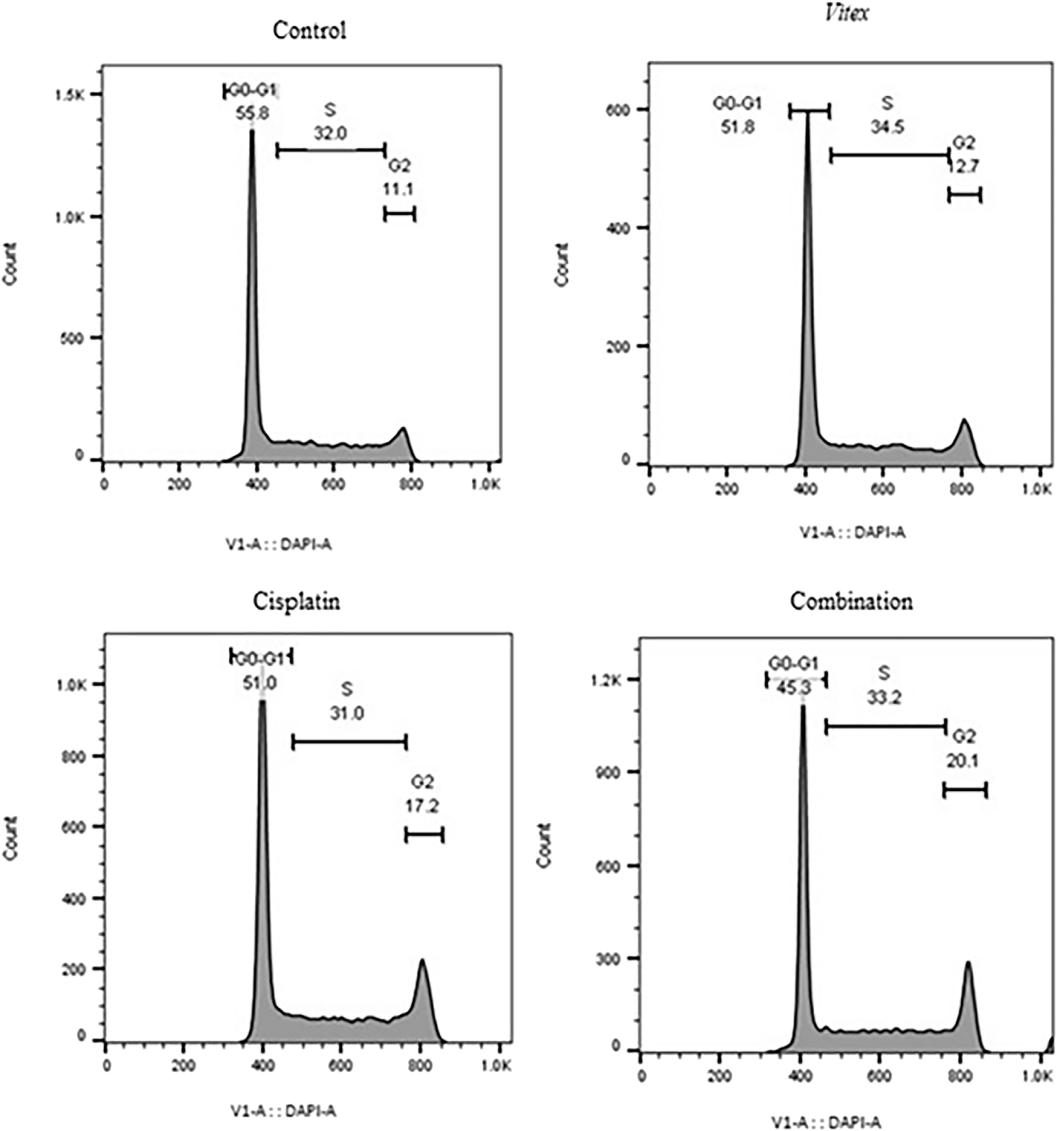
The effect of the *Vitex* extract combined with Cisplatin on the cell cycle in HeLa cells, resulting in cell cycle arrest at the G2-M phase.

**FIGURE 6:**
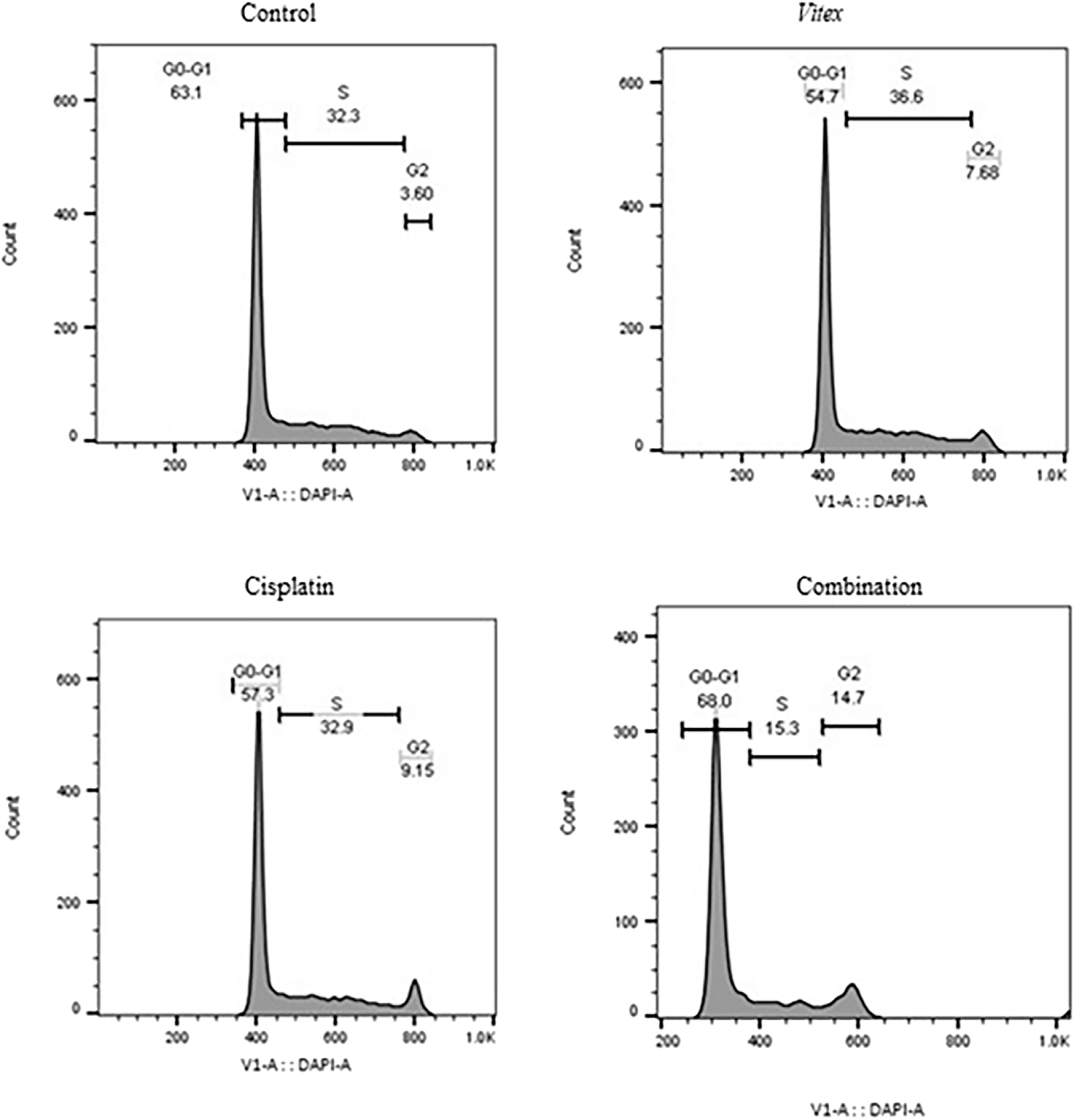
The effect of the Vitex extract combined with Cisplatin on the cell cycle in CaSki cells, leading to cell cycle arrest in the sub-G1 phase.

To better understand the impact of the combined treatment on cell division, the distribution of cell cycles in both cell lines was examined. The findings revealed that CaSki cells underwent an arrest in the G0-G1 phase, while HeLa cells showed arrest predominantly in the G2-M phase after treatment (Figures 5, 6). Following combined treatment with *V. pseudo-Negundo* and Cisplatin (p < 0.0001), a significant increase in cell populations was noted within the G2-M phase, moving from 3.60% to 14.7% for CaSki cells and from 11.1% to 20.1% for HeLa cells, relative to controls. This indicates that the combination therapy enhanced the presence of HeLa cells in the G2-M phase, thereby effectively promoting cell cycle arrest at this juncture (Figure 5). In addition, CaSki cells were also observed to be arrested in the G0-G1 or sub-G1 phase (Figure 6).

### 3.4 ROS production was inhibited by *Vitex pseudo*-*negundo* in combination with cisplatin

**FIGURE 7:**
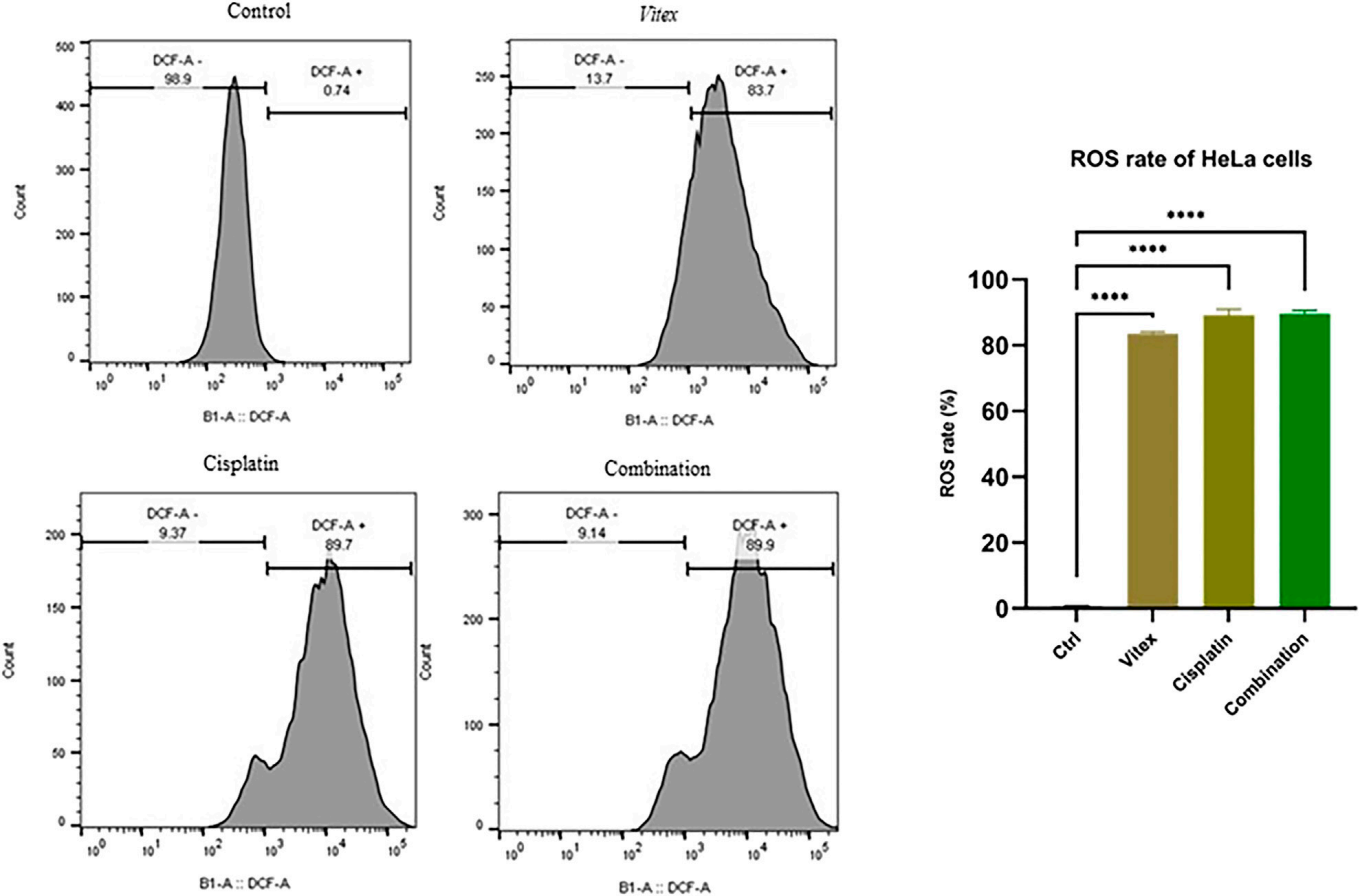
The influence of the Vitex and Cisplatin combination on ROS levels in HeLa cells. Cells in exponential growth were treated with the specified concentrations, and ROS levels were measured via flow cytometry, showing ROS (DCF) levels (%) in relation to the control (*p < 0.05, **p < 0.01, ***p < 0.001, ****p < 0.0001).

**FIGURE 8:**
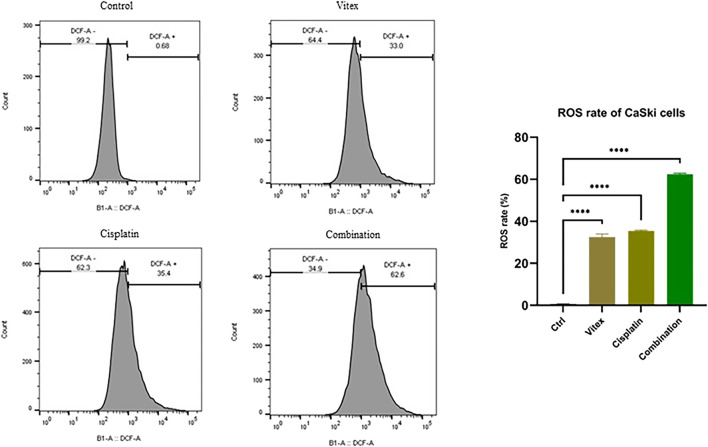
The influence of the Vitex and Cisplatin combination on ROS levels in CaSki cells. Cells in exponential growth were treated with the specified concentrations, and ROS levels were measured via flow cytometry, showing ROS (DCF) levels (%) in relation to the control (*p < 0.05, **p < 0.01, ***p < 0.001, ****p < 0.0001).

To investigate whether the combined treatment with *V. pseudo-Negundo* and Cisplatin affected intracellular ROS levels in cervical cancer cells, measurements were taken after 24 h of either combination therapy or monotherapy. The results demonstrated a significant increase in intracellular ROS levels (DCF) in the combination therapy group compared to controls (p < 0.0001). Notably, in the HeLa cells, ROS levels were found to rise from 0.74% in controls to 89.9% following combination treatment, whereas in CaSki cells, levels increased from 0.68% to 62.6% (Figures 7, 8). Overall, there were no statistically meaningful differences in ROS production among the monotherapy groups (Cisplatin: 89.7%, *V. pseudo-Negundo*: 83.7%) compared to the combination group (89.9%); however, each exhibited significant variations when contrasted with the control (p< 0.0001) (Figure 7). Additionally, the highest ROS production was seen in the CaSki cells treated with both agents, peaking at 62.6%, followed by monotherapy with Cisplatin (35.4%) and *V. pseudo-Negundo* (33%) (Figure 8).

### 3.5 Stemness features in cervical cancer cells were decreased by *Vitex pseudo*-*negundo* fraction in combination with cisplatin

**FIGURE 9:**
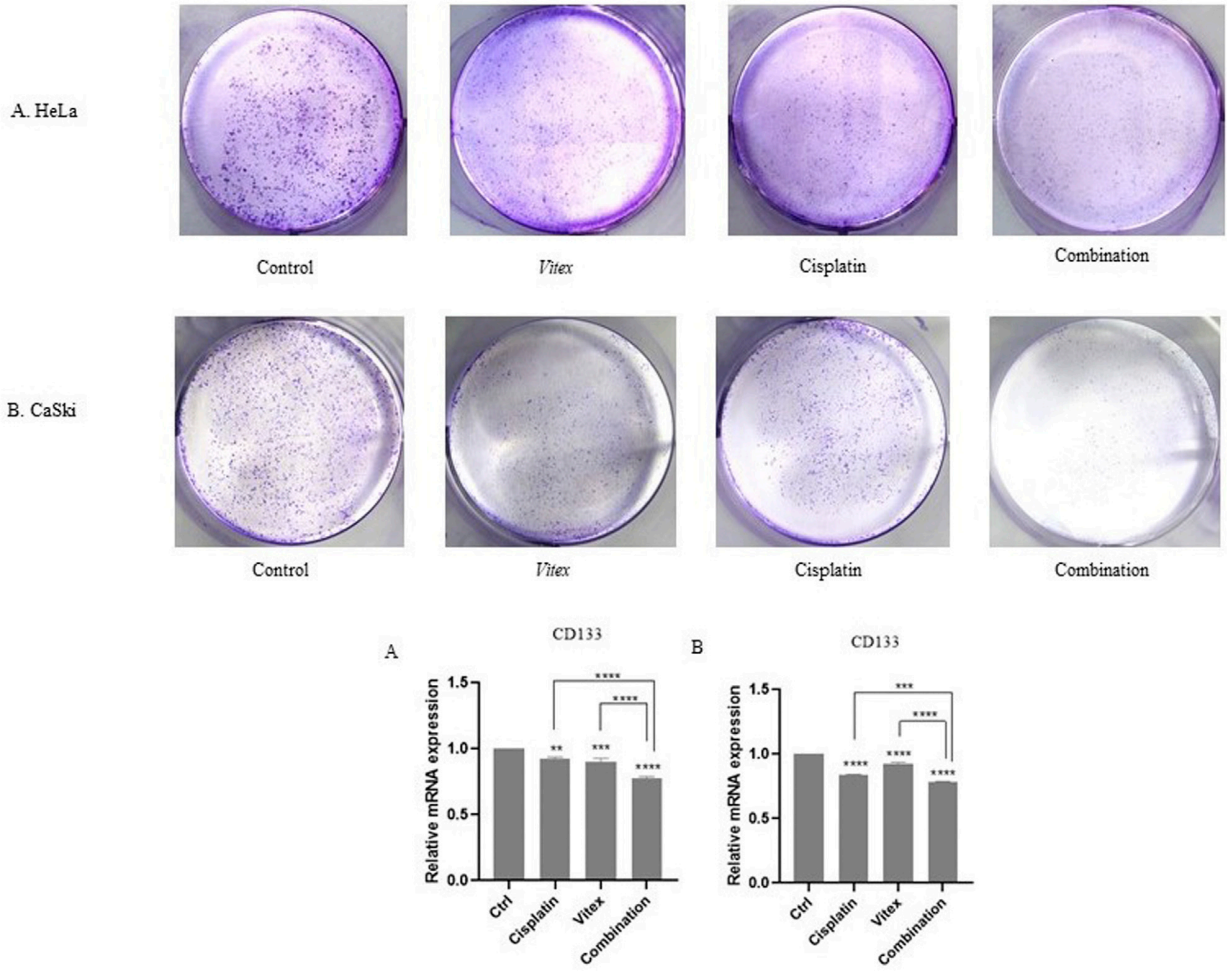
The effect of the *Vitex* and Cisplatin combination on stemness characteristics. **(A)** In HeLa cells, the number of colonies greatly decreases in the combination group when compared to the control. **(B)** A similar decrease in colony numbers is observed in the CaSki cell line with combination treatment. Changes in the stem cell marker CD_133_ are noted in the combination group (*p < 0.05, **p < 0.01, ***p < 0.001, ****p < 0.0001).

To assess the influence of *V. pseudo-Negundo* extract combined with Cisplatin on the stemness traits of cervical cancer cells, a colony formation assay was conducted. Treatments involving both CaSki and HeLa cell lines with the dual agents resulted in a marked reduction in colony formation, particularly notable in the combination group when compared to controls (Figures 9A, B). Treatments with either *V. pseudo-Negundo* or Cisplatin alone also exhibited declines in colony numbers compared to controls, but the combination showed a more substantial impact. To support these findings, RT-PCR analysis was utilized to investigate the expression of stemness-related genes like CD133. Results revealed a significant disparity in CD133 expression levels between the combination treatment group and controls (p < 0.0001) for both cell lines. While reductions in CD133 were noted across all treatment groups, no significant differences were observed among those groups.

### 3.6 Cervical cancerous cells’ motility and related genes have been inhibited by *Vitex pseudo*-*negundo* -cisplatin combination

**FIGURE 10:**
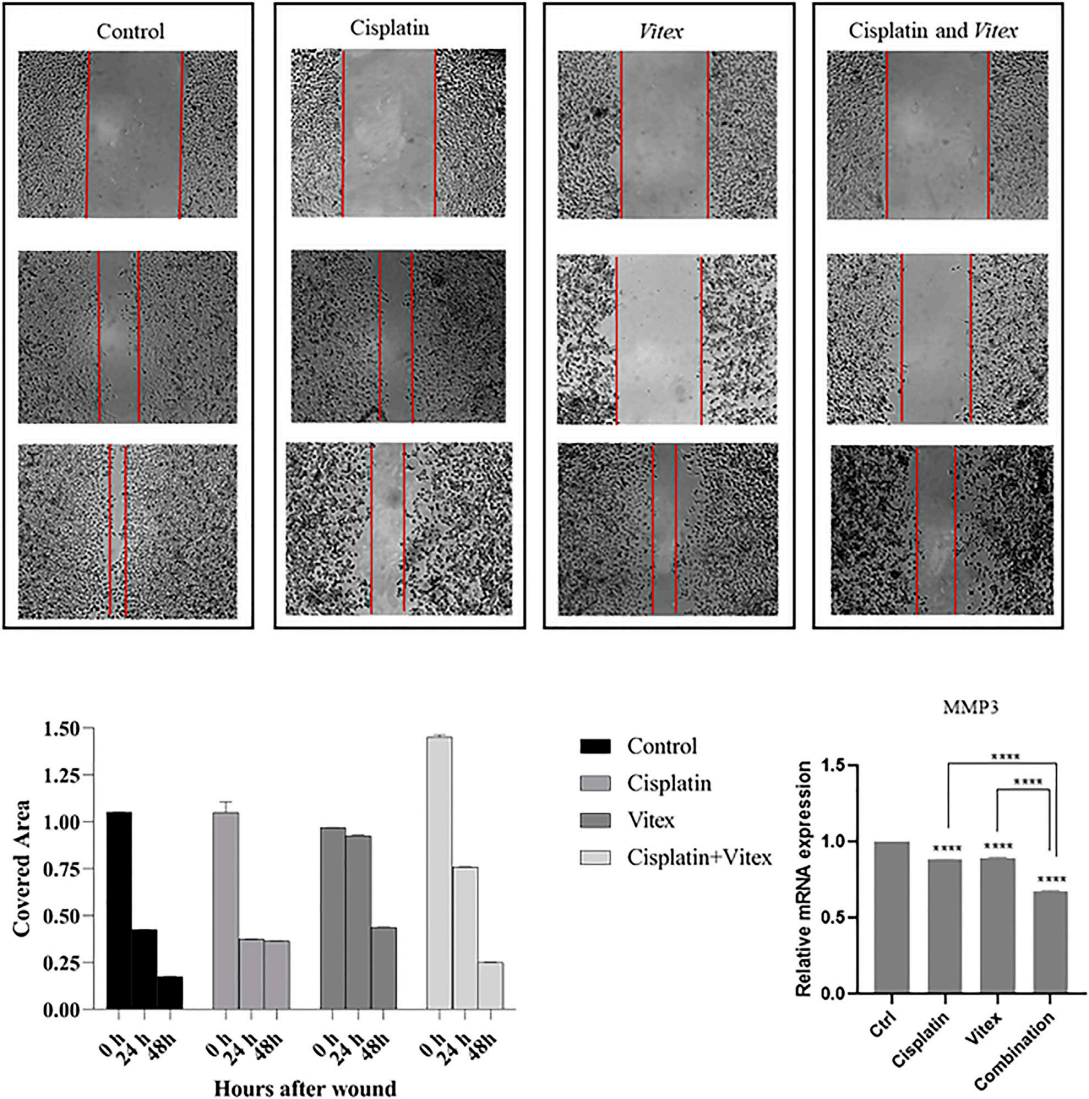
The effect of the *Vitex* and Cisplatin combination on migration and motility in HeLa Cell. After 48 h, this combination results in a significant reduction of motility characteristics compared to control groups. **(A)** Shows covered area by cells after 48 h treatment in which groups. **(B)** Shows alteration of MMP3 expression and compression of it in treatment groups and control group (*p < 0.05, **p < 0.01, ***p < 0.001, ****p < 0.0001).

**FIGURE 11:**
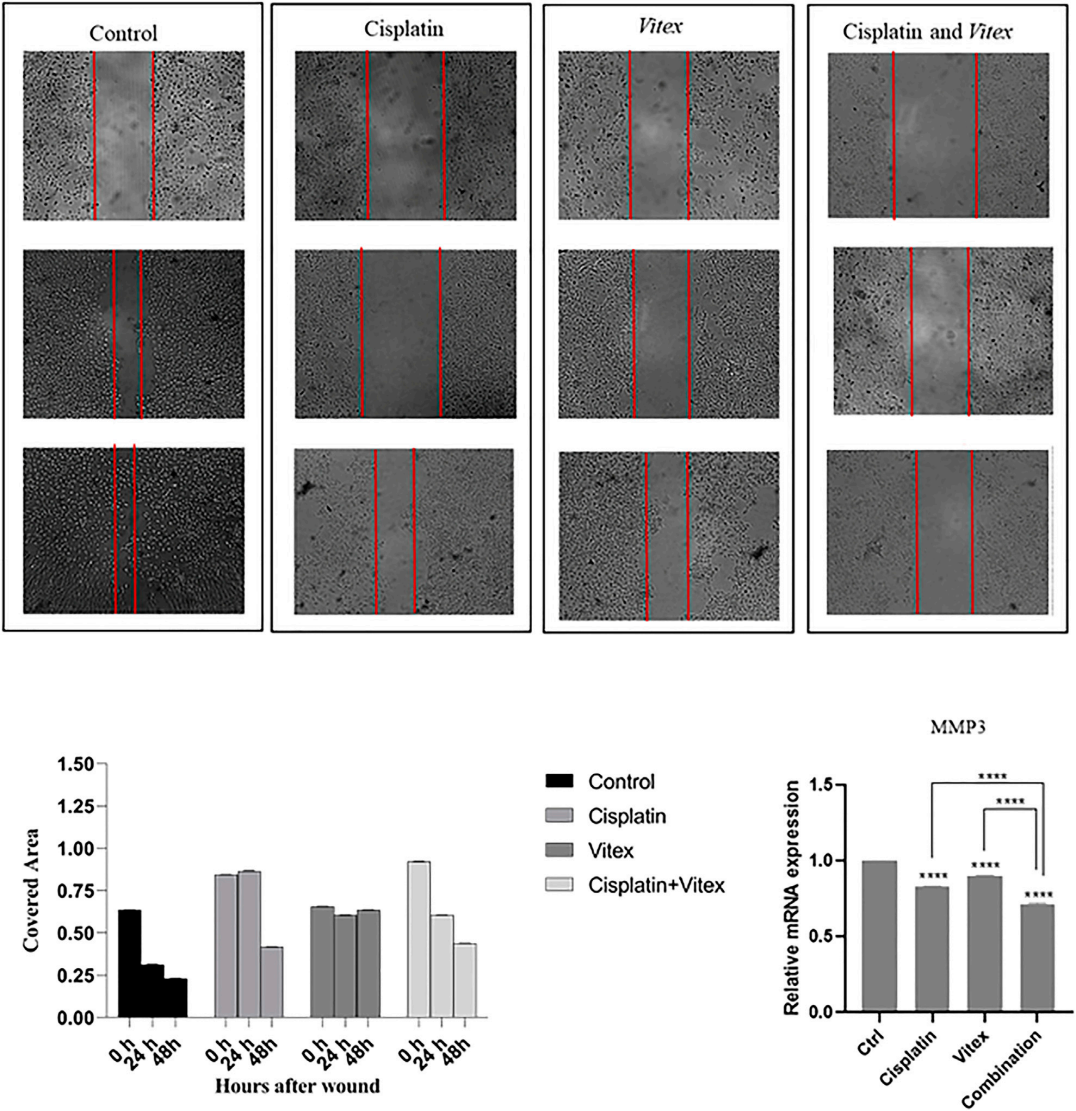
The effect of the *Vitex* and Cisplatin combination on migration and motility in CaSki Cell. After 48 h, this combination results in a significant reduction of motility characteristics compared to control groups. **(A)** Shows covered area by cells after 48 h treatment in which groups. **(B)** Shows alteration of MMP3 expression and compression of it in treatment groups and control group (*p < 0.05, **p < 0.01, ***p < 0.001, ****p < 0.0001).

The wound healing assay results indicated that individual treatments with Cisplatin or *V. pseudo-Negundo* had minimal effects on cervical cancer cell migration. In contrast, the synergistic application of both agents resulted in a significant reduction in the migration of CaSki and HeLa cells following 48 h of treatment compared to controls (Figures 10, 11). Further analysis into the effects of combination treatment on cellular motility employed RT-PCR techniques to evaluate MMP-3 gene expression. This analysis confirmed that the combination of *V. pseudo-Negundo* and Cisplatin effectively reduced MMP-3 levels in both cell lines (p < 0.0001) (Figures 10B, 11B). Although each monotherapy presented reductions in migration and MMP-3 expression, the combination treatment yielded far greater reductions than Cisplatin alone (p < 0.0001), demonstrating significant efficacy even at lower Cisplatin doses.

## 4 Discussion

In this study, we explored the effects of combining *V. pseudo-Negundo* extraction with Cisplatin for cervical cancer (CC) therapy. Our findings indicate that *V. pseudo-Negundo* extract significantly inhibits the growth and proliferation of tumor cells through its growth-inhibitory properties. Specifically, the methanolic extract of *V. pseudo-Negundo* demonstrated a growth inhibitory impact on HeLa and CaSki cell lines at concentrations of 150 μg/mL and 200 μg/mL, with IC_50_ values of 77.49 μg/mL and 81.47 μg/mL, respectively. Notably, the IC_50_ values were higher for CaSki cells than HeLa cells, likely due to the former’s resistance to treatment. Additionally, we observed that the Cisplatin combination with botanical drug remedies enhances the sensitivity of HeLa cells to lower doses of Cisplatin (Madhulika Singh et al., 2013). This suggests that certain plant extracts can improve the response of cancer cells that are resistant to Cisplatin, thereby enhancing the overall efficacy of the treatment (Dasari et al., 2022; Liu et al., 2020). For instance, Erdogan et al. demonstrated that flavonoids could increase the sensitivity of CD44^+^ prostate cancer cells to Cisplatin (Erdogan et al., 2017). In contrast, Li et al. noted similar effects in ovarian cancer cells treated with platinum-based therapies (Li et al., 2014).

Cisplatin is a widely utilized chemotherapeutic agent for various cancers, functioning by disrupting DNA repair mechanisms, causing DNA damage, and promoting apoptosis in cancer cells. However, its effectiveness is often hampered by drug resistance, as well as a range of side effects. Consequently, combination therapies involving Cisplatin and other agents have emerged as a strategy to overcome resistance and mitigate toxicity (Dasari and Bernard Tchounwou, 2014). Herbal medicines in combination therapies have resulted in long-lasting and more potent therapeutic results. Furthermore, they might shield the healthy tissues and trigger sensitivity to chemotherapy and radiotherapy, as well as suppress tumor metastasis and recurrence. Considering the unique characteristics, low side effects, availability, and ease of access, botanical drugs from different regions around the world have been explored, in particular for cancer research (Zulfiker et al., 2017). For instance, the consumption of flavonoids as a part of the *V. pseudo-Negundo* plant leads to cancer prevention and increased sensitivity in Cisplatin-resistant cases, and as a result, promotes Cisplatin treatment. In combined treatments with Cisplatin and flavonoid-rich plants, flavonoids have been associated with increased effectiveness in disrupting the cellular oxidative system, inducing mitochondrial dysfunction, and ultimately leading to apoptosis (Kachadourian et al., 2007). Consistent with our findings, we observed that combining *V. pseudo-Negundo* with Cisplatin resulted in higher levels of reactive oxygen species (ROS) and more pronounced apoptotic responses via a mitochondrial-dependent pathway. Moreover, since Cisplatin is known to induce nephrotoxicity, flavonoids have shown protective effects against this side effect (Casanova et al., 2021). Specifically, they can prevent kidney cell damage by inhibiting apoptosis through the inactivation of p53, mitogen-activated protein kinase (MAPK), and protein kinase B (AKT) signaling pathways (Ju et al., 2015; Wang et al., 2018).

A study conducted by Lui, N et al., in 2018, on *V. negundo L* found that its metabolic, VB1 (vitexin metabolic 1), can induce apoptosis by increasing the expression of pro-apoptotic Bax and decreasing the anti-apoptotic Bcl-2, thereby leading to cell cycle arrest at the G2/M phase in melanoma cells (Liu et al., 2018).

Our data also confirmed that the *V. pseudo-Negundo* can promote cell cycle arrest at the G2-M phase in HeLa cell lines, whereas Cisplatin causes the cell cycle arrest in the G0-G1 phase. Additionally, following treatment with Cisplatin and *V. pseudo-Negundo* fraction, cell cycle arrest in CaSki cell line is comparable to that in the HeLa cell line; however, the rate of cell cycle arrest in the G0-G1 phase remains greater than that in the G2-M phase in the CaSki cell lines. These suggest a synergistic effect for the *V. pseudo-Negundo* and Cisplatin combination. In this regard, Meng et al. reported that Cyclin B1 and CDK1 proteins are significantly reduced after treatment of prostate cancer cells, which results in cell cycle arrest in the G2/M phase (Meng et al., 2012).

Moreover, we observed that monotherapy with *V. pseudo-Negundo* extraction significantly induced apoptosis at high concentrations while inhibiting apoptosis at low concentrations in both cell lines. With that context, we observed that combination therapy enables more effectiveness at lower dosages of *V. pseudo-Negundo* than monotherapy in both cell lines.

Furthermore, our results indicated that the methanolic extraction of *V. pseudo-Negundo* promotes apoptosis through downregulating Bcl-2-related genes and upregulating Bax. Furthermore, the high apoptotic levels were associated with a significant upregulation in the expression level of caspase-3/8/9 in both cell lines, suggesting that apoptosis induction occurs through the caspase-dependent mitochondrial pathway following the combination therapy of *V. pseudo-Negundo* extraction and Cisplatin. Lui et al. also highlighted that changes in gene expression of p53 significantly alter apoptosis and cell cycle pathways (Liu et al., 2018). Thus, we hypothesize that the observed apoptosis rates in our study may hinge on p53 induction, subsequently promoting caspase gene expression and apoptosis through the mitochondrial pathway. Moreover, Khan et al., noted that increased levels of ROS could lead to oxidative damage and DNA damage, ultimately modulating Bcl-2 and reducing mitochondrial membrane integrity, which triggers mitochondrial-dependent apoptosis (Khan et al., 2012).

Furthermore, ROS generation following the use of specific chemotherapeutic agents, can effectively destroy tumor cells through promoting apoptosis. Consequently, it is useful to consider effective ways to achieve significant synergy by combining agents with similar ability to alter redox conditions. Cisplatin is capable of producing ROS that leads to oxidative stress (Poveda et al., 2007; Serkies and Jassem, 2004). While increased ROS can facilitate cancer progression, excessive accumulation leads to apoptosis induction (Gorrini et al., 2013). As a result, to promote the induction of cell death as an anticancer strategy, it is suggested to increase the level of ROS (Raj et al., 2011; Wang et al., 2015; Hang et al., 2018). It has been reported that medicinal metabolites extracted from plants can lead to the induction of intracellular ROS and subsequently DNA damage and increased apoptosis (Hang et al., 2018; Yang et al., 2017).

Here, we demonstrated that *V. pseudo-Negundo* can increase the toxic effect of cisplatin through promoting ROS generation, thereby promoting apoptosis in CC cells. It has been shown that *V. pseudo-Negundo* can enhance the cytotoxic effects of Cisplatin by promoting ROS generation, resulting in increased apoptosis in cervical cancer cells. ROS facilitates the migration of immune cells to damaged tissues and plays a role in infection control during the inflammatory response (Gulumian et al., 2018). Furthermore, ROS promotes the proliferation of keratinocytes, endothelial cells, and fibroblasts, potentially leading to apoptosis and tissue damage (Dunnill et al., 2017). As suggested in this study, elevated ROS levels post-combination treatment was associated with enhanced apoptosis through a mitochondria-dependent pathway in Cisplatin-resistant cells. Liu et al. similarly found that VB1 enhances intracellular ROS accumulation, resulting in DNA damage and increased apoptosis alongside G2/M phase cell cycle arrest in vemurafenib-resistant melanoma cells (Liu et al., 2018).

In addition to apoptosis, which is a critical aspect of cancer progression, we assessed other hallmarks of cancer progression through this experiment. Metastasis is one the most pivotal processes in cancer progression, and MMPs appear to a play critical role in both metastasis and apoptosis processes (Mannello et al., 2005). It has been demonstrated that Cisplatin can suppress cell migration through targeting MMPs (Urso et al., 2010; Montiel et al., 2009). With that context, we established that the Cisplatin- *V. pseudo-Negundo* combination therapy might decrease the expression level of MMPs such as MMP-3 in CC cells.

In addition, significant morphological changes have appeared in CaSki and HeLa cells following *V. pseudo-Negundo* treatment. The combination treatment persuaded a notable number of CC cells to adopt a floating and round-shaped morphology. In this regard, it is speculated that *V. pseudo-Negundo* extract leads to interference in the normal physiological conditions of HeLa and CaSki cells. Moreover, the effect of Cisplatin on morphological alterations has been previously demonstrated in different cells (Gonçalves et al., 2006). Also, the results of the colony formation test indicated that the number of colonies decreased significantly after 14 days compared to the initial days following combination therapy in both cell lines. In other words, the number of colonies in combination groups compared to monotherapy and control groups decreased dramatically, while the colony numbers are not significant between treatment groups (p < 0.0001).

In addition to the prominent anticancer activity of Cisplatin through induction of mitochondrial apoptosis, the high side effects and frequent chemoresistance results in therapeutic failure (Galluzzi et al., 2012). Here, we introduced the ethanolic extraction of the *V. pseudo-Negundo* plant as a promising candidate for combination therapy with Cisplatin in CC treatment. We demonstrated that *V. pseudo-Negundo* has a lethal and antioxidant effect on CaSki and HeLa cancerous cells. Our data also suggested that this natural metabolic exhibited synergistic effects with Cisplatin and can increase cervical cancerous cell features such as apoptosis, ROS production, colony formation, and cell motility suggesting a novel approach for CC treatment.

## 5 Conclusion

In recent decades, preclinical studies have indicated the interaction between chemotherapy drugs and medicinal plant extracts as a therapeutic strategy for many malignancies. Improving the effectiveness of treatment while reducing side effects is one of the aims of combination therapy. Despite the advantages of combined treatments, they may also have disadvantages (Déciga-Campos et al., 2022). So far, the combination therapy of Cisplatin and Paclitaxel is considered a standard chemotherapeutic regimen for the treatment of recurrent or metastatic CC. However, the overall response rate and the median overall survival of patients receiving this regimen are unsatisfactory. In addition, either intrinsic or acquired resistance to Cisplatin can seriously compromise the efficacy of this widely used drug. This underscores the urgent need to introduce novel metabolites that can overcome this resistance, and increase efficacy while exerting minimal cytotoxicity. In this regard, natural metabolites appear to be promising for the treatment of malignancies including CC. Here, we established that the ethanolic extraction of *V. pseudo-Negundo* in combination with Cisplatin can increase its efficacy for the treatment of cervical cancer, suggesting a novel potential for further investigations and offering promise for future clinical therapeutic methods. According to the conducted studies, it has been reported that natural products can prevent the toxicity caused by Cisplatin in different parts of the body, such as the liver, kidney, and nervous system. As a result, the combination therapy of Cisplatin with natural products leads to a reduction in the side effects caused by Cisplatin. Therefore, it is suggested that formulations based on the natural metabolites of Cisplatin be considered and developed as a novel treatment method to prevent the progression of malignancy.

## 6 Limitations

Unfortunately, the number of studies and reports published in our area of study is very limited. Reported studies varied significantly according to the type of cell line, which may have affected the results. In this study, we focused on the main extract; however, it would be preferable to examine the individual active metabolites in the methanolic extract of the *V. pseudo-Negundo* plant in future clinical studies. Also, the lack of access to latest techniques and technologies such as HPLC, NMR, and lack of time for researchers had been influenced. Furthermore, future studies should investigate other genes involved in cellular mechanisms.

## Data Availability

The original contributions presented in the study are included in the article/supplementary material, further inquiries can be directed to the corresponding author.
